# Bidirectional transitions of frailty states among middle-aged and older adults: a longitudinal cohort analysis using a multi-state Markov model based on the China Health and Retirement Longitudinal Study

**DOI:** 10.1093/geroni/igaf095

**Published:** 2025-09-09

**Authors:** Ye Tong, Yiling Teng, Yujie Zhang, Changming Huang, Weiliang Liao, Baicheng Wan, Shaohui Zong, Gaofeng Zeng

**Affiliations:** Department of Spine Osteopathic, The First Affiliated Hospital of Guangxi Medical University, Nanning, China; Department of Spine Osteopathic, The First Affiliated Hospital of Guangxi Medical University, Nanning, China; Department of Spine Osteopathic, The First Affiliated Hospital of Guangxi Medical University, Nanning, China; Department of Spine Osteopathic, The First Affiliated Hospital of Guangxi Medical University, Nanning, China; Department of Spine Osteopathic, The First Affiliated Hospital of Guangxi Medical University, Nanning, China; Department of Spine Osteopathic, The First Affiliated Hospital of Guangxi Medical University, Nanning, China; Department of Spine Osteopathic, The First Affiliated Hospital of Guangxi Medical University, Nanning, China; Department of Orthopedic Surgery, Wuming Hospital of Guangxi Medical University, Nanning, China; Department of Nutrition and Food Hygiene, School of Public Health, Guangxi Medical University, Nanning, China

**Keywords:** State transition, Sojourn time, Recovery probability

## Abstract

**Background and Objectives:**

Frailty is a dynamic syndrome increasing older adults’ vulnerability to adverse outcomes. Longitudinal data on frailty transitions and their influencing factors remain limited. We aimed to examine bidirectional frailty transitions among middle-aged and older adults using a multi-state Markov (MSM) model.

**Research Design and Methods:**

Data were obtained from four waves (2011-2018) of the China Health and Retirement Longitudinal Study (CHARLS), including 15 763 participants aged ≥45 years. Frailty was assessed using a 32-item frailty index. MSM models estimated transition probabilities, mean sojourn times, and covariate effects. Additional analyses examined age- and gender-specific patterns and included an age × gender interaction term.

**Results:**

Baseline prevalence of robust, pre-frail, and frail states was 44.3%, 39.4%, and 16.3%, respectively. Within one year, pre-frail participants had probabilities of 18.0% reverting to robust and 19.7% progressing to frail states. At five years, these probabilities were 23.4% and 33.4%, respectively, with mortality increasing to 19.7%. Older age increased frailty progression and mortality risks but reduced recovery likelihood. Notably, significant age × gender interactions were observed for transitions from pre-frail to robust and from frail to death. Men showed higher recovery rates but greater frailty-related mortality than women. Urban residency, higher education, and marriage were protective, while smoking and alcohol increased frailty risk.

**Discussion and Implications:**

Frailty among middle-aged and older Chinese adults demonstrates substantial bidirectional transitions, indicating notable opportunities for intervention and prevention. Age, gender, socioeconomic status, and lifestyle behaviors are key modifiable determinants influencing frailty progression and recovery. Public health strategies prioritizing targeted screening and preventive interventions—particularly among vulnerable groups—could effectively mitigate frailty progression, promote recovery, and improve overall population health outcomes.

Innovation and Translational Significance This study provides robust evidence of the dynamic nature and reversibility of frailty among middle-aged and older adults. By identifying modifiable demographic and lifestyle risk factors associated with frailty transitions, our findings enable clinicians and public health policymakers to design targeted interventions aimed at preventing or delaying frailty progression. Ultimately, translating these insights into clinical practice could significantly enhance health outcomes and quality of life for aging populations.

## Introduction

Frailty, an age-associated clinical condition characterized by progressive deterioration across multiple physiological systems and heightened vulnerability to stressors, represents a substantial and increasing public health challenge globally.^1^ Frail individuals often experience adverse outcomes such as falls, hospitalization, disability, and increased mortality, significantly impacting their quality of life and imposing considerable economic burdens on healthcare systems.^2^ Although traditionally viewed primarily through the lens of physical impairment, contemporary consensus highlights frailty’s multidimensional nature, encompassing physical, cognitive, psychological, and social domains.^3^ Emerging longitudinal evidence emphasizes that frailty is a dynamic, bidirectional condition where individuals transition between states of robustness, pre-frailty, and frailty over time, underscoring potential windows of opportunity for interventions to reverse or slow frailty progression.^4–6^ Given the rapidly aging global population, particularly in low- and middle-income countries, understanding frailty transitions and identifying modifiable determinants remain critical priorities for clinical practice, healthcare policy, and preventive strategies.^7^

Multi-state Markov (MSM) models have become increasingly prominent in longitudinal health research because of their unique ability to rigorously characterize the complex and dynamic nature of health trajectories. Unlike traditional analytic methods that typically capture only unidirectional or static outcomes, MSMs explicitly model bidirectional transitions between multiple discrete health states and estimate state-­specific sojourn times, thereby reflecting the true clinical course of conditions such as frailty. These models enable researchers to quantify not only the probabilities of progression, regression, and stability across health states, but also to assess the effects of covariates on each possible transition pathway.^8^ Importantly, MSMs can estimate both instantaneous transition intensities (hazard rates) and cumulative transition probabilities over different time intervals, while accommodating recurrent transitions and competing absorbing states such as death. Furthermore, they are well-suited to handling interval-censored data, which is common in cohort studies with periodic assessments, and allow for the incorporation of transition-specific covariate effects.^9^^,^^10^ Previous studies have effectively applied MSM models to characterize dynamic transitions across various health states, such as sarcopenia and mild cognitive impairment, highlighting these models’ strengths in quantifying the probabilities of progression, regression, and stability, as well as examining the influence of relevant covariates on these transitions.^11–13^ Recent large-scale cohort studies in Western high-income countries have consistently demonstrated the dynamic and reversible nature of frailty using MSM models. Analyses of more than two million adults in England revealed strong associations between frailty transitions and age, deprivation, and ethnicity, and emphasized the accelerating burden of frailty with advancing age.^14^ Evidence from the English Longitudinal Study of Ageing (ELSA) showed that wealth, education, area deprivation, and marital status are powerful determinants of both frailty-free and frail life expectancy, with the most advantaged individuals living more than a decade longer without frailty than the most disadvantaged.^15^ Irish cohort data further illustrated that frailty states are not fixed: significant proportions of older adults transition from frail or prefrail to more robust states over time, highlighting important windows for intervention.^16^^,^^17^ Taken together, this growing body of evidence demonstrates that MSMs are exceptionally well-suited to capture the inherently dynamic and reversible nature of frailty, providing a robust methodological framework for investigating the complexity of frailty transitions in aging populations. However, the vast majority of longitudinal research on frailty dynamics—and particularly on bidirectional transitions—has focused on older adults in Western, high-income settings. There remains a critical gap in our understanding of frailty transitions in low- and middle-income countries (LMICs), where demographic profiles, cultural contexts, health behaviors, and access to healthcare differ substantially.

To address this gap, we used data from the nationally representative China Health and Retirement Longitudinal Study (CHARLS) to examine the probabilities and determinants of frailty state transitions among Chinese middle-aged and older adults using MSM models. We hypothesized that frailty transitions in this population would be dynamic and bidirectional and that demographic and behavioral factors may be associated with differing risks of progression or recovery. As China experiences one of the most rapid demographic shifts toward population aging in the world, the growing prevalence of frailty poses significant challenges to the country’s health system, social care infrastructure, and long-term resource planning. The findings from this study will provide critical evidence to inform targeted strategies for frailty prevention and healthy aging, not only in China but also in other low- and middle-­income countries facing similar demographic and public health challenges.

## Methods

### Study design and participants

**Figure 1.**
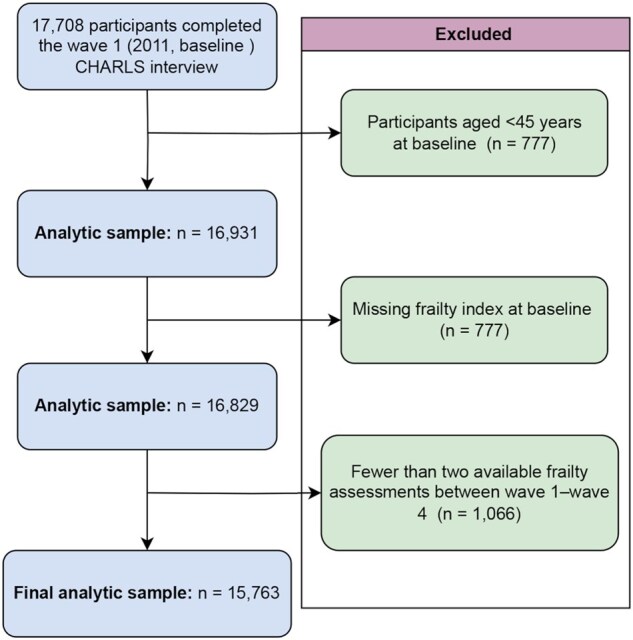
Flowchart of participant selection for the current study.

This study used data from the China Health and Retirement Longitudinal Study (CHARLS), a nationally representative longitudinal survey of Chinese adults aged 45 years or older. CHARLS used a multistage stratified probability-proportional-­to-size sampling method covering 150 counties/districts and 450 villages/urban communities across 28 provinces of mainland China. Details on the study design and sampling framework are publicly accessible through the official CHARLS website (http://charls.pku.edu.cn/). For this analysis, data were drawn from four survey waves: Wave 1 (2011), Wave 2 (2013), Wave 3 (2015), and Wave 4 (2018). A total of 17 708 participants completed the Wave 1 (2011) interview. Although CHARLS primarily targets adults aged 45 years and older, a small number of respondents younger than 45 years were present in the baseline sample. Therefore, we excluded 777 participants who were aged <45 years at baseline, resulting in 16 931 eligible participants aged ≥45 years. Among these, 777 were further excluded due to missing frailty index data at baseline, yielding 16 829 participants with available frailty index. Finally, 1066 individuals lacking at least two available frailty assessments across Waves 1-4 were excluded. Consequently, the final analytic sample included 15 763 participants for baseline frailty and transition analyses (Figure 1).

### Definition and assessment of frailty status

Frailty status was defined using the Rockwood frailty index (FI), an established cumulative-deficit measure widely used in clinical and epidemiological studies.^18^ The FI quantifies frailty as the proportion of health deficits present in an individual across various domains—including chronic conditions, functional impairments, cognitive status, depressive symptoms, and psychosocial aspects—thus reflecting an individual’s biological aging and vulnerability to adverse outcomes. The item selection process followed both international methodological recommendations and best practices for FI construction in cohort studies. Specifically, we applied the 10-step approach outlined by Theou et al.,^19^ which emphasizes comprehensive selection of age-related health deficits from multiple physiological domains, exclusion of variables with excessive missingness or limited variability, standardization of deficit coding, confirmation of age association, minimization of high inter-item correlations, and sufficient domain coverage (≥30 items). Guided by these criteria, our final 32-item set was informed by, and is directly comparable to, several high-quality frailty indices previously validated in the CHARLS cohort, as reported in recent peer-reviewed studies using similar deficit-accumulation approaches.^20–22^ Nearly all core items from these studies were included, with several additional deficits added to enhance coverage and maximize data completeness. All selected variables were available at each survey wave, exhibited acceptable data quality, and collectively represent the multidimensional health status most relevant to frailty in Chinese older adults.

In our study, the FI comprised 32 items encompassing baseline health conditions, chronic diseases, physical functional impairments, depressive symptoms, and cognitive status. Specifically, each of the first 31 items was dichotomized based on predefined cutoff values, with deficits scored as “1” (presence of the health deficit) or “0” (absence of the health deficit). Notably, item 32 (cognitive function score) was measured continuously, ranging from 0 to 1, with higher scores indicating poorer cognitive performance. Detailed descriptions and coding rules for all 32 items are provided in Supplementary Table 1. To maintain indicator accuracy while addressing missing data and maximizing the use of available information, we allowed a maximum of 10% missingness (ie, ≤3 items) per participant. Individuals with more than three missing items (>10%) had their FI coded as missing and were excluded from analyses involving FI. For participants with three or fewer missing items, the FI was calculated as the sum of the participant’s health deficits divided by the total number of non-missing items. For example, if a participant had 30 valid (non-missing) items, the FI was computed as the sum of health deficits present divided by 30. Higher FI values reflect greater frailty severity. Participants were then categorized into frailty states according to established FI cutoff values commonly applied in clinical practice and previous literature: robust (FI ≤0.10), pre-frail (0.10 < FI < 0.25), and frail (FI ≥0.25). Frailty status assessments were performed at each available CHARLS wave (Wave 1 to Wave 4), enabling longitudinal analyses of state transitions over time.

### Outcome definition and transition states

**Figure 2.**
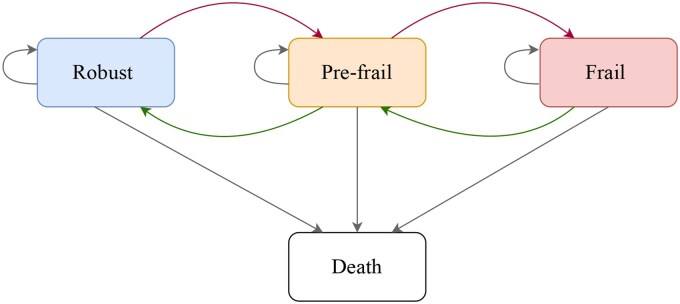
Schematic representation of the multi-state Markov model used to estimate transitions among frailty states. The model includes three transient states—robust, pre-frail, and frail—and one absorbing state (death). Bidirectional transitions are allowed between adjacent transient states, while death is modeled as an absorbing state that cannot be exited. Arrows indicate all possible transitions among states considered in the model. The structure captures both progression and potential recovery across frailty states, as well as direct transitions to death from any frailty level.

The primary outcome of interest in this study was the bidirectional transition among frailty states, assessed longitudinally across four CHARLS waves. Frailty transitions were modeled using three transient frailty states—robust, pre-frail, and frail—along with an absorbing state of death. In our MSM model, transitions were strictly limited to adjacent states: that is, robust could only transition to pre-frail (and vice versa), pre-frail could only transition to robust or frail, and frail could only transition to pre-frail. Additionally, transitions from any frailty state to death (the absorbing state) were permitted, but once participants entered the absorbing state, no further transitions were possible (Figure 2). Direct transitions between non-adjacent transient states (eg, robust directly to frail or frail directly to robust) were not allowed in the model structure. Participants were categorized according to their frailty status at each assessment wave. Transition probabilities and intensities between states were subsequently estimated using ­continuous-time MSM models. This modeling approach accounts explicitly for the dynamic, bidirectional nature of frailty progression and provides insights into the natural course of frailty states over time.

### Covariates

Covariates included demographic variables such as age, sex, marital status (married/partnered, other marital status), residency (rural or urban), and education level (below middle school, middle school, high school and above). Health-related behaviors assessed were smoking status (never smoker, ever smoker) and alcohol consumption (never drinker, ever drinker). All covariates were collected consistently across CHARLS Waves 1-4 through standardized face-to-face questionnaires administered by trained interviewers. Body mass index (BMI) and physical activity were excluded from analysis due to substantial missing data or measurement unavailability (BMI was not measured in Wave 4; physical activity had a missing rate exceeding 50%). Missing covariate data were handled through multiple imputation procedures, minimizing potential bias and ensuring robust analyses.

### Statistical analysis

Continuous-time MSM models were used to evaluate transitions among frailty states and to estimate transition intensities and probabilities over the study period. The MSM approach provides a rigorous statistical framework to characterize bidirectional transitions among transient states (robust, pre-frail, and frail) and unidirectional transitions toward the absorbing state (death). In the MSM model, transitions from one state to another depended exclusively on the current state (Markov assumption). Transition intensity *q_ij_* represents the instantaneous hazard of moving from state *i* to state *j*. The transition intensity matrix Q for the current MSM model is defined as follows (robust, pre-frail, frail, and death states were coded numerically as 1, 2, 3, and 4, respectively):


$$Q=\left(\begin{array}{cccc} -(q_{12}+q_{14}) & q_{12} & 0 & q_{14}\\ q_{21} & -(q_{21}+q_{23}+q_{24}) & q_{23} & q_{24}\\ 0 & q_{32} & -(q_{32}+q_{34}) & q_{34}\\ 0 & 0 & 0 & 0 \end{array}\right)$$

The probabilities of transitioning between states within specified time intervals (1-year, 3-year, and 5-year) were calculated using the MSM model. Initially, an MSM model without covariates was constructed to estimate baseline transition intensities. Subsequently, a multivariable MSM model was developed by incorporating covariates to adjust for potential confounding. Hazard ratios (HRs) with corresponding 95% confidence intervals (CIs) were derived for each covariate, reflecting their effects on frailty-state transitions. Moreover, transition probabilities stratified by age and sex subgroups were estimated to explore potential heterogeneity in frailty dynamics. To assess potential effect modification, an additional MSM was fitted, including an age × gender interaction term, allowing the evaluation of whether the impact of age on frailty transitions differed between men and women. To further characterize the dynamics of frailty, we calculated the mean sojourn time. The mean sojourn time for each transient state was defined as the expected average duration an individual remained in a given state before transitioning to other states. Mathematically, the mean sojourn time in state *i* was computed as the reciprocal of the sum of all transition intensities from state *i* to other states, expressed as follows (*q_ij_* represents the transition intensity from state *i* to state *j*):$\text{Mean}\;\text{Sojourn}\;\text{Time}\;\text{in}\;\text{state}\;i=\frac{1}{\sum_{j\neq i}q_{ij}}$

Additionally, we calculated the total duration of stay in frail transient states across the follow-up period, enabling comprehensive interpretation of long-term frailty burden within the population. Statistical analyses were performed using R version 4.4.0 (http://www.r-project.org/), specifically using the MSM package.^23^ Missing data were addressed using multiple imputation by chained equations (MICE), ensuring robustness and minimizing bias from missing observations.^24^ Statistical significance was set at a two-sided *p*-value of <.05.

## Results

### Baseline and follow-up characteristics

Baseline characteristics of the 15 763 participants are presented in Table 1. At baseline, 6989 (44.3%) participants were classified as robust, 6208 (39.4%) as pre-frail, and 2566 (16.3%) as frail. The mean age of the study population was 59.1 years (SD 9.7), with a significant gradient observed across frailty groups: frail participants were older on average (65.2 years), followed by pre-frail (59.6 years) and robust individuals (56.5 years) (*P* < .001). Women comprised 51.1% of the cohort, with the proportion of women increasing progressively from robust (43.1%) to pre-frail (55.5%) and frail (62.2%) groups (*P* < .001). The majority of participants (67.2%) had an education level below middle school; this proportion was highest among frail individuals (84.8%) and lowest among robust individuals (57.3%) (*P* < .001). Most participants were married or partnered (87.4%), with a higher prevalence among robust participants (92.1%) and a lower prevalence in the frail group (76.9%) (*P* < .001). Regarding residency, 61.3% of participants resided in rural areas, with the proportion increasing from robust (57.7%) to frail (68.9%) (*P* < .001). For health behaviors, 40.3% reported ever smoking and 42.0% reported ever drinking alcohol. Frail participants were less likely to report a history of smoking (34.5%) or drinking (35.2%) compared with robust (44.4% and 46.3%, respectively) and pre-frail participants (38.1% and 39.9%, respectively) (both *PP* < .001). All group differences were statistically significant (*P* < .001), indicating substantial heterogeneity in demographic, socioeconomic, and behavioral characteristics across frailty categories at baseline. Over the course of follow-up, the distribution of frailty status and deaths changed substantially (Supplementary Table 2). The proportion of participants classified as frail increased progressively, reaching 24.5% by Wave 4, while the percentage of robust individuals declined to 30.1%. Mortality also rose over time, with 2.8% of participants deceased by Wave 2, 3.8% by Wave 3, and 6.1% by Wave 4.

**Table 1.** Baseline characteristics of participants for baseline frailty status analyses.

Characteristic	Overall (%) *N *= 15 763	Frailty status			*p*
		Robust (%) *n *= 6989 (44.3%)	Pre-frail (%) *n *= 6208 (39.4%)	Frail (%) *n *= 2566 (16.3%)	
Gender, *n* (%)					<.001
Female	8052 (51.08)	3013 (43.11)	3444 (55.48)	1595 (62.16)	
Male	7711 (48.92)	3976 (56.89)	2764 (44.52)	971 (37.84)	
Education, *n* (%)					<.001
Below middle school	10 587 (67.16)	4002 (57.26)	4410 (71.04)	2175 (84.76)	
Middle school	3241 (20.56)	1795 (25.68)	1173 (18.89)	273 (10.64)	
High school or above	1935 (12.28)	1192 (17.06)	625 (10.07)	118 (4.60)	
Age in years, mean (SD)	59.09 (9.67)	56.45 (8.65)	59.55 (9.30)	65.16 (10.29)	<.001
Marital status, *n* (%)					<.001
Other marital status	1987 (12.61)	554 (7.93)	839 (13.51)	594 (23.15)	
Married or partnered	13 776 (87.39)	6435 (92.07)	5369 (86.49)	1972 (76.85)	
Residency, *n* (%)					<.001
Rural	9658 (61.27)	4032 (57.69)	3858 (62.15)	1768 (68.90)	
Urban	6105 (38.73)	2957 (42.31)	2350 (37.85)	798 (31.10)	
Smoke, *n* (%)					<.001
Never smokers	9408 (59.68)	3886 (55.60)	3841 (61.87)	1681 (65.51)	
Ever smokers	6355 (40.32)	3103 (44.40)	2367 (38.13)	885 (34.49)	
Drink, *n* (%)					<.001
Never drinkers	9149 (58.04)	3753 (53.70)	3733 (60.13)	1663 (64.81)	
Ever drinkers	6614 (41.96)	3236 (46.30)	2475 (39.87)	903 (35.19)	

### Dynamics of frailty state transitions

**Figure 3.**
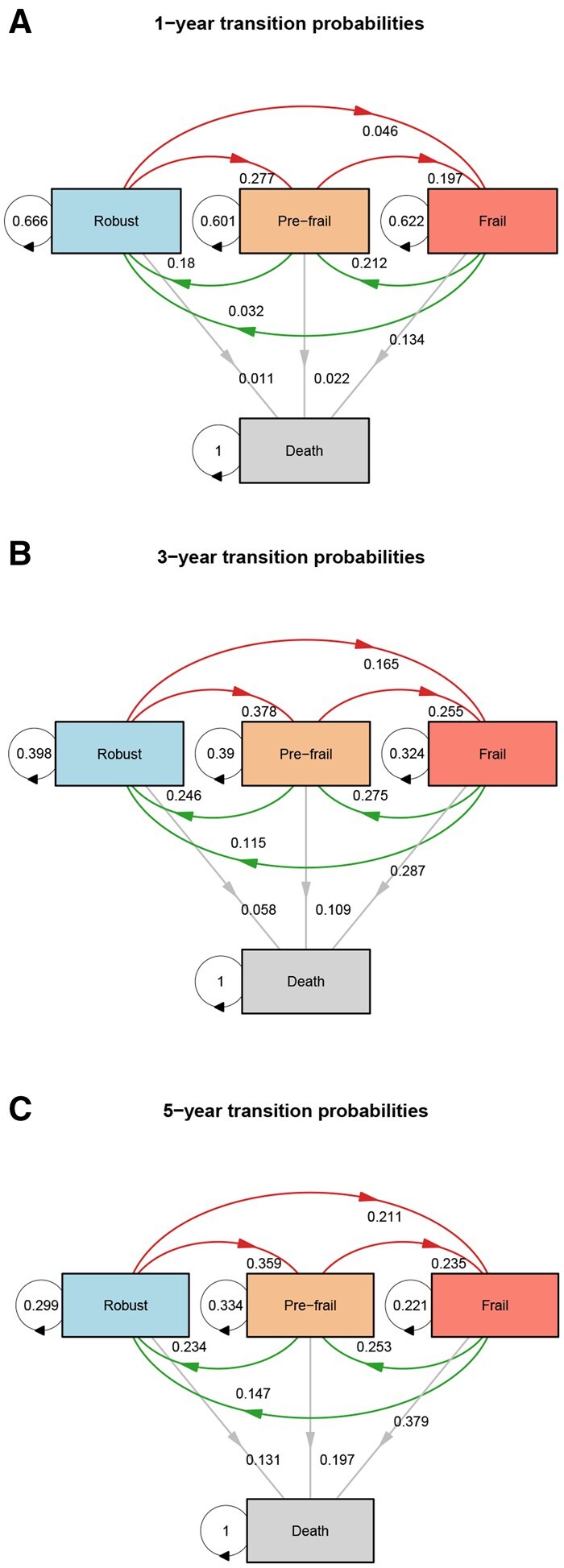
Estimated transition probabilities between frailty states over 1-, 3-, and 5-year intervals. Panels (A), (B), and (C) display the 1-, 3-, and 5-year transition probabilities, respectively, among the three transient frailty states (robust, pre-frail, and frail) and the absorbing state (death). Directed arrows are annotated with the corresponding transition probabilities; looped arrows indicate the probability of remaining in the same state. Worsening transitions are defined as movements from robust to pre-frail and from pre-frail to frail; recovery transitions are defined as movements from pre-frail to robust and from frail to pre-frail; transitions to death originate from any state and terminate in the absorbing state.

The analysis of frailty state transitions among middle-aged and older Chinese adults reveals a dynamic and bidirectional nature over 1-, 3-, and 5-year intervals (Figure 3). Within a 1-year period, individuals classified as pre-frail had probabilities of 18.0%, 60.1%, 19.7%, and 2.2% for reverting to robust status, remaining pre-frail, progressing to frailty, and transitioning to death, respectively. After 3 years, these probabilities changed notably, with 24.6% recovering to robust status, 25.5% maintaining pre-frail status, 30.9% deteriorating to frailty, and 10.9% experiencing death. At 5-year follow-up, pre-frail individuals had probabilities of 23.4% reverting to robust, 23.5% remaining pre-frail, 33.4% progressing to frailty, and 19.7% dying, indicating a progressive increase in adverse outcomes over longer durations. For participants initially identified as frail, the 1-year probabilities of reverting to robust status, improving to pre-frail, remaining frail, and dying were 3.2%, 21.2%, 62.2%, and 13.4%, respectively. Over a 3-year period, the corresponding probabilities shifted to 11.5% for reverting to robust status, 27.5% improving to pre-frail, 32.4% maintaining frailty, and a substantial increase in mortality risk to 28.7%. At the 5-year mark, frail individuals exhibited further dynamic shifts: the probabilities of reverting to robust status increased slightly to 14.7%, improving to pre-frail status was 25.3%, remaining frail decreased to 22.1%, whereas the probability of death significantly rose to 37.9%. These findings highlight the fluidity of frailty states, underscoring both the considerable potential for recovery and the increasing risk of progression to frailty and death over time.

### Covariate-adjusted associations with frailty state transitions

After adjusting for covariates, significant associations between demographic and lifestyle factors and frailty state transitions were identified (Figure 4, Supplementary Table 3). Age was strongly associated with frailty dynamics; each additional year significantly increased the risk of progression from robust to pre-frail (HR: (after all HR’s and CI’s) 1.03, 95% CI 1.02-1.03), pre-frail to frail (HR 1.04, 95% CI 1.04-1.05), and transition to death from robust (HR 1.04, 95% CI 1.00-1.08), pre-frail (HR 1.03, 95% CI 1.00-1.06), and frail states (HR 1.07, 95% CI 1.06-1.07). Conversely, increasing age reduced the likelihood of reverting from pre-frail to robust (HR 0.99, 95% CI 0.98-0.99) and frail to pre-frail states (HR 0.98, 95% CI 0.97-0.98). Gender-specific differences were observed; compared with women, men had a higher likelihood of reverting from pre-frail to robust (HR 1.17, 95% CI 1.04-1.31) and frail to pre-frail (HR 1.26, 95% CI 1.09-1.45), but a significantly lower risk of progressing from robust to pre-frail (HR 0.73, 95% CI 0.66-0.80), pre-frail to frail (HR 0.79, 95% CI 0.71-0.88), and notably higher risk of death from frail states (HR 2.00, 95% CI 1.69-2.37). Urban residency significantly reduced the risk of progression from pre-frail to frail states (HR 0.84, 95% CI 0.78-0.90). Married or partnered individuals had a lower mortality risk from frail states compared with individuals of other marital statuses (HR 0.83, 95% CI 0.72-0.96). Educational attainment exhibited protective effects; individuals with middle school education showed reduced progression from robust to pre-frail (HR 0.89, 95% CI 0.83-0.96) and pre-frail to frail (HR 0.81, 95% CI 0.73-0.89), but increased mortality risk from frail states (HR 1.30, 95% CI 1.06-1.59). Higher education (high school or above) further decreased the risk of progression from robust to pre-frail (HR 0.75, 95% CI 0.68-0.83), pre-frail to frail (HR 0.63, 95% CI 0.55-0.73), and improved the likelihood of recovery from frail to pre-frail (HR 0.80, 95% CI 0.64-0.99). Smoking significantly increased risks of deterioration from robust to pre-frail (HR 1.12, 95% CI 1.03-1.23), pre-frail to frail (HR 1.19, 95% CI 1.07-1.31), and mortality from frail states (HR 1.20, 95% CI 1.03-1.40). Alcohol consumption (“ever drinkers”) was associated with a notably increased mortality risk among pre-frail individuals (HR 1.73, 95% CI 1.03-2.91). Incorporation of the age × gender interaction term into the MSM model identified statistically significant effects for the transitions from pre-frail to robust (HR 0.99, 95% CI: 0.98-1.00) and from frail to death (HR 0.98, 95% CI: 0.97-0.99). For all other transitions, the age × gender interaction was not statistically significant (all *P *> .05). The main effects of age and gender on frailty transitions are reported in Supplementary Table 4.

**Figure 4. Hazard ratios (HRs) of covariates associated with transitions between frailty states based on the multivariable multi-state Markov model. Each column denotes a specific state transition (including transitions to death), and each row represents a covariate. Points indicate HRs and horizontal bars show 95% confidence intervals. Associations are considered statistically significant when the 95% confidence interval does not include 1.0 (two-sided p < .05).**
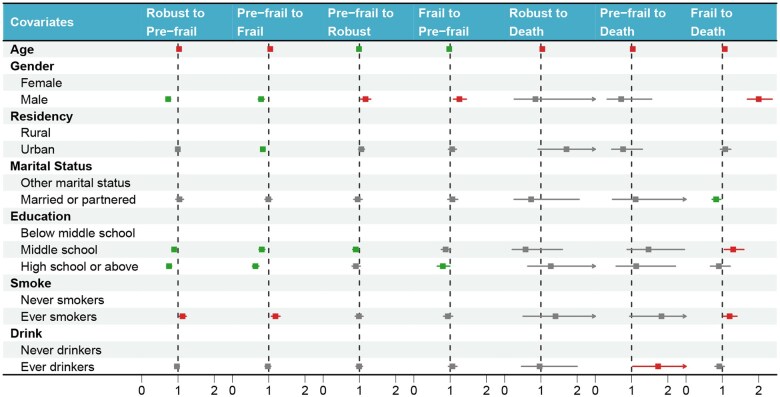


### Subgroup analysis

**Figure 5.**
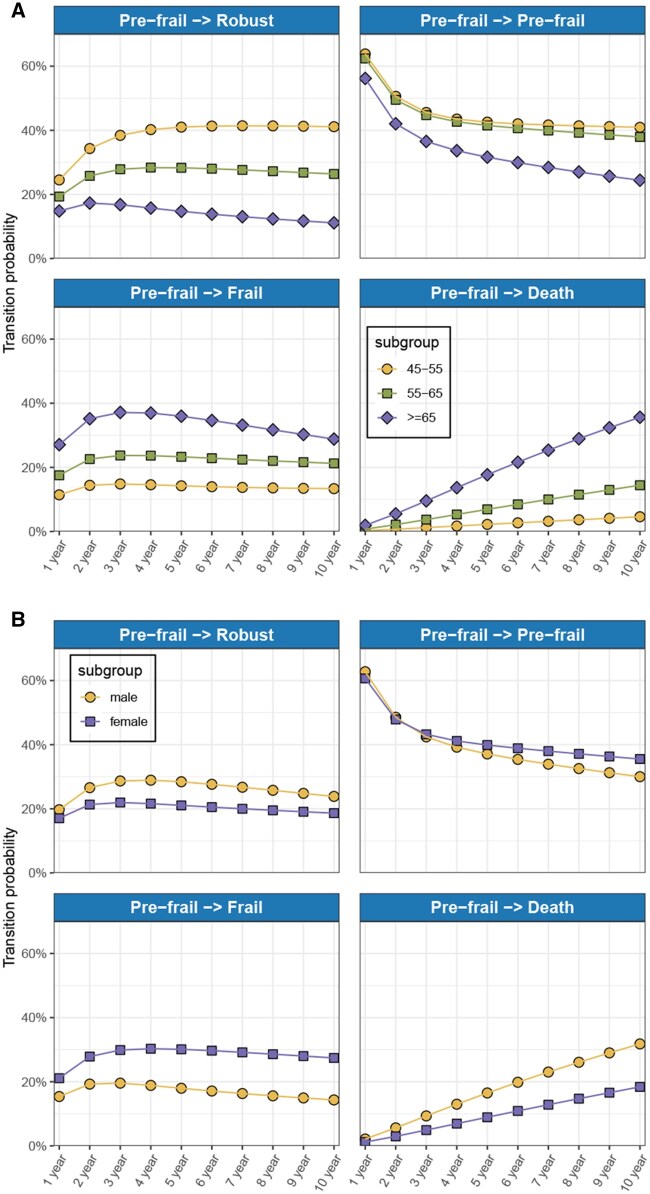
Subgroup-specific transition probabilities from the pre-frail state over a 10-year period. (A) Age-stratified transition probabilities from the pre-frail state to robust, frail, and death states, as well as remaining pre-frail, estimated over a 10-year interval. (B) Gender-stratified transition probabilities from the pre-frail state, showing differences between male and female participants across the same four transition trajectories.

Considering the significant joint effects of age and gender on both deterioration and recovery of frailty states identified in previous analyses, we conducted further multivariable MSM subgroup analyses stratified by age groups (45-54, 55-64, and ≥65 years) and gender. Transition probabilities, specifically focusing on pre-frail state transitions, were recalculated within these subgroups (Figure 5). The age-stratified subgroup analyses revealed distinct trends in transition probabilities from the pre-frail state across three age groups. Overall, younger participants (aged 45-54 years) exhibited consistently higher probabilities of reverting from pre-frail to robust states over time, whereas older participants (≥65 years) demonstrated markedly lower recovery rates. Conversely, the likelihood of progression from pre-frail to frail or death increased substantially with advancing age, with the oldest age group showing the highest transition probabilities toward frailty and mortality throughout the observation period. Additionally, the probability of remaining in the pre-frail state decreased over time across all age groups, with older individuals again showing the steepest declines. The gender-stratified subgroup analyses demonstrated notable differences in transition probabilities from the pre-frail state between men and women. Overall, men consistently exhibited higher probabilities of recovery from pre-frail to robust states across the observation period compared with women. In contrast, women displayed a substantially higher probability of progressing from pre-frail to frail states over time. Regarding mortality, men showed a progressively increasing probability of transitioning from pre-frail to death, surpassing the risk observed among women, particularly at longer follow-up durations. The probability of remaining in the pre-frail state decreased similarly over time for both genders.

**Figure 6.**
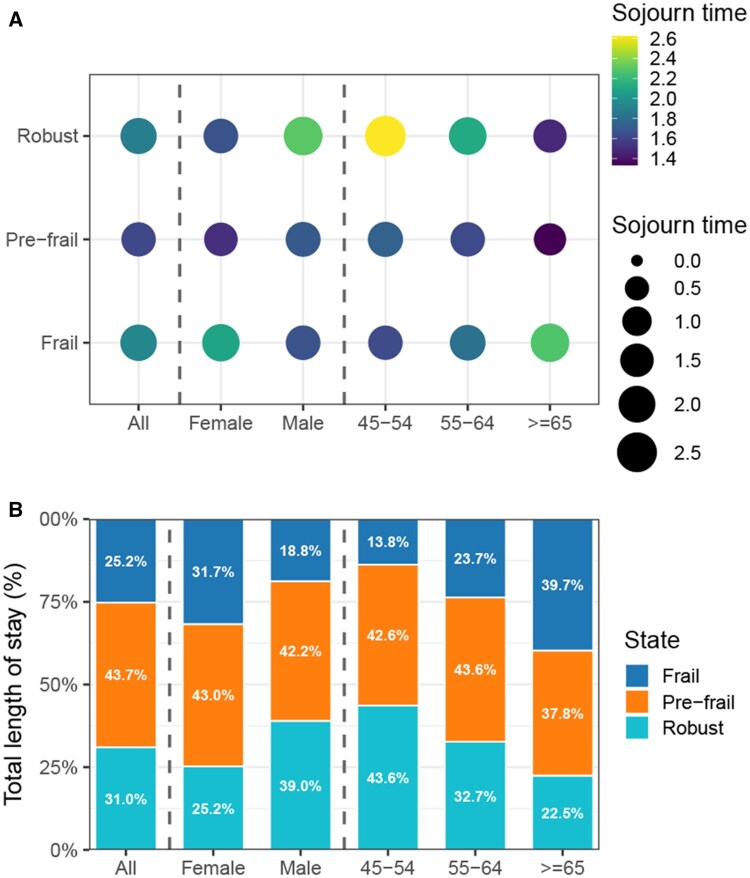
Estimated mean sojourn time and predicted total length of stay in frailty states stratified by age and gender subgroups. (A) Mean sojourn time represents the expected average duration spent in each transient frailty state prior to transitioning into the next state, presented separately for the overall cohort as well as stratified by gender and age groups. (B) Predicted total length of stay in each frailty state expressed as proportions of the total time prior to death over a 10-year interval.

Analysis of the mean sojourn time—the average duration individuals spent in each transient frailty state—revealed notable differences across age and gender subgroups (Figure 6A). Overall, younger individuals (aged 45-54 years) exhibited longer mean sojourn times in the robust state, reflecting greater stability compared with older individuals (≥65 years), who experienced shorter durations in robust states and correspondingly longer durations in frail states. Gender differences, while less pronounced, were still evident: men generally remained in the robust state slightly longer than women, whereas women tended to spend marginally more time in pre-frail and frail states.

The predicted total length of stay in each frailty state prior to death also varied considerably by age and gender (Figure 6B). Collectively, individuals spent the largest proportion of their time in the pre-frail state (43.7%), followed by robust (31.0%) and frail states (25.2%). Women spent relatively more time in frail states (31.7%) compared with men (18.8%), whereas men spent a larger proportion of time in robust states (39.0%) compared with women (25.2%). With advancing age, there was a substantial decline in the proportion of time spent in the robust state, accompanied by a corresponding increase in the duration spent in frail states, highlighting the significant impact of aging on frailty trajectories.

## Discussion

In this longitudinal cohort study of middle-aged and older adults drawn from the nationally representative CHARLS dataset, we observed dynamic and bidirectional transitions across frailty states over 1-, 3-, and 5-year intervals, highlighting both deterioration and substantial potential for recovery among participants. Frailty dynamics were strongly influenced by demographic factors such as age and gender, as well as lifestyle behaviors including smoking and alcohol consumption. Specifically, advancing age markedly increased risks for frailty progression and mortality, while significantly reducing the likelihood of recovery to healthier states. Gender-specific differences were also prominent, with women demonstrating higher risks of transitioning toward frailty, whereas men exhibited greater probabilities of recovery from pre-frail to robust states. Additionally, individuals residing in urban areas, those with higher educational attainment, and married or partnered individuals exhibited reduced risks of frailty progression or death, whereas smoking and alcohol consumption were associated with increased frailty progression and mortality risks. Subgroup analyses further illustrated that younger participants (aged 45-54 years) spent substantially longer durations in healthier states, with a greater capacity for recovery, whereas older participants (≥65 years) faced higher cumulative frailty burdens and shorter durations in robust states. Prior longitudinal studies have documented frequent bidirectional changes in frailty status among older populations, indicating substantial potential for both deterioration and recovery. For instance, Travers et al.^25^ reported that structured interventions involving physical activity and dietary protein significantly reversed frailty status among community-dwelling older adults, underscoring the modifiable nature of frailty trajectories. Furthermore, studies have confirmed that frailty status, assessed via electronic frailty indices or phenotype-based approaches, is predictive of adverse clinical outcomes, including higher mortality risk and postoperative complications.^26^^,^^27^ Consistent with these observations, our results highlight that frailty transitions significantly correlate with mortality and further extend these insights by explicitly quantifying the probabilities of recovery and deterioration across different follow-up intervals. Our analysis identified specific demographic and lifestyle factors influencing frailty transitions, corroborating findings from recent large-scale epidemiological studies. Hanlon et al.^28^ demonstrated clear associations between socioeconomic indicators—including educational attainment and marital status—and frailty prevalence and transitions, with lower socioeconomic positions associated with higher frailty burden and reduced probability of recovery. Similarly, smoking and alcohol consumption have been repeatedly linked to higher frailty risk, consistent with the inflammatory and metabolic mechanisms identified in biomarker-based studies.^29^ These findings collectively underscore the importance of targeted prevention and early intervention strategies that account for age, gender, and modifiable risk factors to effectively manage frailty trajectories and promote healthier aging. Health education campaigns and community-based frailty screening programs could focus on enhancing health literacy, particularly among individuals with lower educational backgrounds—a strategy shown to delay frailty onset and promote recovery.^30^ Smoking cessation and reduction in harmful alcohol consumption can be integrated into routine geriatric assessments, and primary care providers should be trained to recognize pre-frailty and deliver brief interventions at early stages.^31^ Social and marital support, which was associated with lower frailty progression, could be fostered through structured peer networks, community centers, and mental health resources for older adults.^32^ Collectively, these strategies can be operationalized through multidisciplinary geriatric care pathways and age-friendly community initiatives.

Furthermore, emerging evidence underscores the importance of gender differences in frailty dynamics, consistently reporting a higher prevalence of frailty among women, accompanied by intricate gender-specific variations in patterns of recovery and mortality risk.^33^ Our results substantiate these gender differences, indicating greater frailty progression among women but superior recovery probabilities among men. Despite typically having longer lifespans, women are more likely to experience frailty across diverse populations and measurement methodologies—a phenomenon known as the “sex-frailty paradox.” This paradox highlights the intriguing observation that while women generally bear a higher burden of frailty and display increased susceptibility to health deficits, they paradoxically maintain a survival advantage, exhibiting lower mortality risk than men at comparable levels of frailty.^34^ Multiple biological, behavioral, and psychosocial mechanisms have been proposed to explain these observed sex differences. Biologically, women demonstrate more robust innate and adaptive immune responses, resulting in a heightened pro-inflammatory milieu—often described as “inflamm-­aging”—which accelerates the accumulation of health deficits and the development of frailty, but may also provide enhanced immune surveillance and tissue repair, thereby supporting greater survival.^35^^,^^36^ Hormonal influences further contribute: the decline of estrogen after menopause in women is associated with increased bone loss, muscle wasting, and inflammation, all of which predispose to frailty; conversely, age-related reductions in testosterone among men are linked to accelerated loss of physiological reserve and increased risk of adverse outcomes.^37^ At the molecular level, recent transcriptomic studies have revealed sex-specific aging patterns, with women exhibiting slower molecular aging trajectories but accumulating more chronic, non-lethal deficits, while men are prone to more rapid decline in vital organ systems.^38^ From a clinical perspective, women are more likely to develop disabling but non-fatal chronic conditions—such as osteoporosis, arthritis, and depression—which contribute to elevated frailty scores without necessarily increasing short-term mortality risk.^39^ In contrast, men are at higher risk of acute, fatal events, particularly cardiovascular diseases, which contribute to their lower survival despite lower frailty prevalence.

Socio-behavioral factors also play a role: women are generally more engaged in healthcare utilization, demonstrate greater adherence to medical interventions, and maintain stronger social support networks, all of which are associated with better recovery from frailty and lower mortality.^40^ Conversely, men are more likely to engage in high-risk behaviors such as smoking and excessive alcohol consumption, and are less likely to seek timely medical care, further exacerbating mortality risk independent of frailty status.^41^ Although both men and women benefit from physical activity promotion and nutritional optimization, tailored approaches that address sex-specific vulnerabilities may enhance outcomes further. For example, interventions emphasizing osteoporosis prevention through targeted weight-bearing exercise and calcium-vitamin D supplementation may be especially beneficial for women, while men may derive greater benefit from interventions focused on cardiovascular risk reduction, such as aerobic exercise regimens and targeted pharmacological management.^42^ These insights collectively highlight the necessity of incorporating sex-specific considerations into frailty prevention and management strategies, presenting opportunities to enhance clinical outcomes and improve overall quality of life in older populations.

Methodologically, the application of a MSM model in our study allowed for a detailed examination of state-specific transition probabilities, offering nuanced insights beyond conventional logistic or Cox regression models commonly used in frailty research.^43^ The use of MSM facilitates explicit modeling of transient states and provides comprehensive estimates of sojourn times, advancing the understanding of frailty as a dynamic and reversible health state. Such methodological refinement not only aligns with current best practices recommended by recent systematic reviews but also emphasizes the necessity of using advanced statistical techniques to capture the complexity inherent in frailty trajectories. China’s unprecedented demographic shift toward an aging society is accelerating the burden of frailty and disability among older adults.^44^ Our findings emphasize that frailty is a dynamic and modifiable condition, and highlight the public health imperative for early screening, targeted lifestyle interventions, and integrated care strategies. National policy initiatives have expanded primary care and insurance coverage, but further innovation in community-based prevention and comprehensive geriatric management is essential to meet the needs of a rapidly growing older population.^45^ Evidence shows that person-centered, function-oriented models—combining health literacy, social engagement, and chronic disease management—can delay frailty onset, reduce disparities, and promote healthy longevity.^46^ As China and other rapidly aging societies confront these challenges, timely and scalable frailty interventions will be critical for public health sustainability and healthy aging at the population level.

The primary strengths of our study include its large-scale, nationally representative cohort from the CHARLS dataset, which provides robust statistical power and generalizability to the broader population of middle-aged and older adults in China. Furthermore, the application of the MSM model allowed us to precisely quantify transition probabilities among frailty states, simultaneously capturing both progression and recovery over multiple follow-up intervals. This approach provides a comprehensive understanding of frailty dynamics that traditional analytical methods may overlook. Additionally, detailed subgroup analyses stratified by age and gender offered valuable insights into demographic-specific frailty trajectories, facilitating the identification of populations most at risk and thus informing targeted preventive interventions. Nevertheless, several limitations must be acknowledged. First, frailty status was assessed based on a constructed frailty index derived from self-reported and objectively measured items, which may introduce reporting bias or measurement errors. Second, although multiple demographic and behavioral covariates were considered, other potentially influential factors—such as nutritional status, detailed physical activity patterns, medication usage, and genetic predispositions—were not included due to data limitations, potentially leading to residual confounding. Third, the observational nature of our study precludes definitive causal inferences regarding the relationships identified. Finally, given that the study population consisted solely of Chinese adults aged 45 and older, the generalizability of our findings to younger individuals or other ethnic populations remains uncertain.

## Conclusion

In this nationally representative longitudinal study using MSM modeling, we demonstrate that frailty transitions among middle-aged and older adults in China are highly dynamic and bidirectional. Within five years, approximately one-quarter (23.4%) of participants initially classified as pre-frail returned to a robust state, whereas one-third (33.4%) progressed to frailty and nearly one-fifth (19.7%) died. Notably, even among frail individuals, meaningful recovery to pre-frail (25.3%) or robust states (14.7%) occurred, accompanied by considerable mortality risk (37.9%), highlighting critical opportunities for intervention. We further identify specific modifiable determinants of these frailty transitions, including a significant age-gender interaction. Older age consistently reduced the likelihood of recovery and increased risks of frailty progression and mortality. Men exhibited greater recovery potential but faced higher frailty-related mortality compared with women. Protective factors included urban residency, higher educational attainment, and marital partnership, whereas smoking and alcohol consumption were associated with increased frailty risks. Given China’s rapidly aging population, our findings emphasize the importance of implementing early frailty screening programs and targeted preventive interventions. Prioritizing modifiable risk factors—particularly among vulnerable subgroups such as older women, rural residents, and individuals with lower socioeconomic status—could effectively reduce frailty progression, enhance recovery, and ultimately improve health outcomes and longevity at the population level.

## Supplementary Material

igaf095_Supplementary_Data

## Data Availability

The data underlying this article are available in the China Health and Retirement Longitudinal Study (CHARLS) at http://charls.pku.edu.cn/. All derived data supporting the findings presented in this study are available from the corresponding author upon reasonable request. All R code snippets necessary for the MSM model construction, including model formulas and parameter settings, are available at https://zenodo.org/records/15761115.
