# Supplementary material for: Bidirectional transitions of frailty states among middle-aged and older adults: a longitudinal cohort analysis using a multi-state Markov model based on the China Health and Retirement Longitudinal Study
Source: Innov Aging. 2025 Sep 9;9(12):igaf095. doi: 10.1093/geroni/igaf095 (PMC12685369; doi:10.1093/geroni/igaf095)
Supplement: igaf095_Supplementary_Data [file igaf095_supplementary_data.docx]

***Innovation in Aging* Supplementary Material: Tong, Teng, Zhang, Huang, Liao, Wan, Zong, & Zeng. Bidirectional transitions of frailty states among middle-aged and older adults: A longitudinal cohort analysis using a multi-state Markov model based on the CHARLS.**

**Supplementary Table 1.** The 32 items used to construct the frailty index

| **No.** | **Description of the 32 items** | **Cut-off value** |
| --- | --- | --- |
| 1 | Self-reported physician diagnosed hypertension | Yes = 1, No = 0 |
| 2 | Self-reported physician diagnosed diabetes | Yes = 1, No = 0 |
| 3 | Self-reported physician diagnosed heart problems | Yes = 1, No = 0 |
| 4 | Self-reported physician diagnosed stroke | Yes = 1, No = 0 |
| 5 | Self-reported physician diagnosed cancer | Yes = 1, No = 0 |
| 6 | Self-reported physician diagnosed arthritis | Yes = 1, No = 0 |
| 7 | Self-reported physician diagnosed chronic lung disease | Yes = 1, No = 0 |
| 8 | Self-reported physician diagnosed asthma | Yes = 1, No = 0 |
| 9 | Self-reported physician diagnosed any emotional, nervous, or psychiatric problems | Yes = 1, No = 0 |
| 10 | Self-reported physician diagnosed memory-related disease | Yes = 1, No = 0 |
| 11 | Self-reported vision problems | Yes = 1, No = 0 |
| 12 | Self-reported hearing problems | Yes = 1, No = 0 |
| 13 | Difficulty with dressing | Yes = 1, No = 0 |
| 14 | Difficulty with bathing or showering | Yes = 1, No = 0 |
| 15 | Difficulty with eating | Yes = 1, No = 0 |
| 16 | Difficulty with getting in and out of bed | Yes = 1, No = 0 |
| 17 | Difficulty with using the toilet | Yes = 1, No = 0 |
| 18 | Difficulty with managing money | Yes = 1, No = 0 |
| 19 | Difficulty with taking medication | Yes = 1, No = 0 |
| 20 | Difficulty with shopping for groceries | Yes = 1, No = 0 |
| 21 | Difficulty with preparing meals | Yes = 1, No = 0 |
| 22 | Difficulty with doing housework | Yes = 1, No = 0 |
| 23 | Mobility: difficulty with walking 100 yards or one block | Yes = 1, No = 0 |
| 24 | Mobility: difficulty with getting up from a chair after sitting for long periods | Yes = 1, No = 0 |
| 25 | Mobility: difficulty with climbing several flights of stairs without resting | Yes = 1, No = 0 |
| 26 | Mobility: difficulty with lifting or carrying weights over 10 pounds/jins | Yes = 1, No = 0 |
| 27 | Mobility: difficulty with picking up a coin from the table | Yes = 1, No = 0 |
| 28 | Mobility: difficulty with stooping, kneeling, or crouching | Yes = 1, No = 0 |
| 29 | Mobility: difficulty with reaching arms above shoulder level | Yes = 1, No = 0 |
| 30 | Self-reported health | Very poor/ Poor/ Fair = 1, Very good/ Good = 0 |
| 31 | Depressive symptoms: CESD-10 questionnaire | CESD-10 >10 = 1, ≤10 = 0 |
| 32 | Cognition: (memory test score + orientation test score) / 14 | Continuous variable (from 0 to 1) |

**Supplementary Table 2.** Number (percentage) of participants by frailty status and wave

|  | **Wave 1** | **Wave 2** | **Wave 3** | **Wave 4** |
| --- | --- | --- | --- | --- |
| Frail | 2566 (16.3%) | 2415 (16.8%) | 2800 (20.3%) | 3238 (24.5%) |
| Pre-frail | 6208 (39.4%) | 5739 (39.9%) | 5722 (41.5%) | 5213 (39.4%) |
| Robust | 6989 (44.3%) | 5820 (40.5%) | 4736 (34.4%) | 3986 (30.1%) |
| Death | 0 | 398 (2.8%) | 529 (3.8%) | 801 (6.1%) |

**Supplementary Table 3.** Hazard ratios (HRs) of covariates associated with transitions between frailty states based on the multivariable multi-state Markov model

| **Transition** | **Covariates** | **Reference** | **HR (95% CI)** | ***p*** |
| --- | --- | --- | --- | --- |
| Pre-frail->Robust | Age | - | 0.99 (0.98 - 0.99) | <0.001 |
| Robust->Pre-frail | Age | - | 1.03 (1.02 - 1.03) | <0.001 |
| Frail->Pre-frail | Age | - | 0.98 (0.97 - 0.98) | <0.001 |
| Pre-frail->Frail | Age | - | 1.04 (1.04 - 1.05) | <0.001 |
| Robust->Death | Age | - | 1.04 (1.00 - 1.08) | 0.042 |
| Pre-frail->Death | Age | - | 1.03 (1.00 - 1.06) | 0.023 |
| Frail->Death | Age | - | 1.07 (1.06 - 1.07) | <0.001 |
| Pre-frail->Robust | Male | Female | 1.17 (1.04 - 1.31) | 0.009 |
| Robust->Pre-frail | Male | Female | 0.73 (0.66 - 0.80) | <0.001 |
| Frail->Pre-frail | Male | Female | 1.26 (1.09 - 1.45) | 0.002 |
| Pre-frail->Frail | Male | Female | 0.79 (0.71 - 0.88) | <0.001 |
| Robust->Death | Male | Female | 0.85 (0.27 - 2.71) | 0.783 |
| Pre-frail->Death | Male | Female | 0.71 (0.33 - 1.55) | 0.394 |
| Frail->Death | Male | Female | 2.00 (1.69 - 2.37) | <0.001 |
| Pre-frail->Robust | Urban | Rural | 1.05 (0.97 - 1.14) | 0.217 |
| Robust->Pre-frail | Urban | Rural | 0.99 (0.93 - 1.06) | 0.846 |
| Frail->Pre-frail | Urban | Rural | 1.06 (0.95 - 1.17) | 0.312 |
| Pre-frail->Frail | Urban | Rural | 0.84 (0.78 - 0.90) | <0.001 |
| Robust->Death | Urban | Rural | 1.71 (0.92 - 3.16) | 0.089 |
| Pre-frail->Death | Urban | Rural | 0.76 (0.45 - 1.30) | 0.32 |
| Frail->Death | Urban | Rural | 1.08 (0.95 - 1.24) | 0.253 |
| Pre-frail->Robust | Married or partnered | Other marital status | 0.95 (0.84 - 1.08) | 0.43 |
| Robust->Pre-frail | Married or partnered | Other marital status | 1.04 (0.94 - 1.15) | 0.458 |
| Frail->Pre-frail | Married or partnered | Other marital status | 1.06 (0.93 - 1.21) | 0.361 |
| Pre-frail->Frail | Married or partnered | Other marital status | 0.99 (0.90 - 1.09) | 0.815 |
| Robust->Death | Married or partnered | Other marital status | 0.73 (0.26 - 2.05) | 0.553 |
| Pre-frail->Death | Married or partnered | Other marital status | 1.11 (0.47 - 2.62) | 0.81 |
| Frail->Death | Married or partnered | Other marital status | 0.83 (0.72 - 0.96) | 0.011 |
| Pre-frail->Robust | Middle school | Below middle school | 0.90 (0.81 - 0.99) | 0.031 |
| Robust->Pre-frail | Middle school | Below middle school | 0.89 (0.83 - 0.96) | 0.003 |
| Frail->Pre-frail | Middle school | Below middle school | 0.88 (0.76 - 1.01) | 0.077 |
| Pre-frail->Frail | Middle school | Below middle school | 0.81 (0.73 - 0.89) | <0.001 |
| Robust->Death | Middle school | Below middle school | 0.58 (0.21 - 1.59) | 0.289 |
| Pre-frail->Death | Middle school | Below middle school | 1.47 (0.87 - 2.46) | 0.147 |
| Frail->Death | Middle school | Below middle school | 1.30 (1.06 - 1.59) | 0.012 |
| Pre-frail->Robust | High school or above | Below middle school | 0.90 (0.79 - 1.02) | 0.11 |
| Robust->Pre-frail | High school or above | Below middle school | 0.75 (0.68 - 0.83) | <0.001 |
| Frail->Pre-frail | High school or above | Below middle school | 0.80 (0.64 - 0.99) | 0.042 |
| Pre-frail->Frail | High school or above | Below middle school | 0.63 (0.55 - 0.73) | <0.001 |
| Robust->Death | High school or above | Below middle school | 1.27 (0.64 - 2.55) | 0.493 |
| Pre-frail->Death | High school or above | Below middle school | 1.13 (0.57 - 2.21) | 0.729 |
| Frail->Death | High school or above | Below middle school | 0.90 (0.67 - 1.22) | 0.508 |
| Pre-frail->Robust | Ever smokers | Never smokers | 0.98 (0.88 - 1.10) | 0.789 |
| Robust->Pre-frail | Ever smokers | Never smokers | 1.12 (1.03 - 1.23) | 0.008 |
| Frail->Pre-frail | Ever smokers | Never smokers | 0.94 (0.82 - 1.07) | 0.331 |
| Pre-frail->Frail | Ever smokers | Never smokers | 1.19 (1.07 - 1.31) | <0.001 |
| Robust->Death | Ever smokers | Never smokers | 1.40 (0.51 - 3.87) | 0.513 |
| Pre-frail->Death | Ever smokers | Never smokers | 1.82 (0.94 - 3.52) | 0.074 |
| Frail->Death | Ever smokers | Never smokers | 1.20 (1.03 - 1.40) | 0.021 |
| Pre-frail->Robust | Ever drinkers | Never drinkers | 0.99 (0.91 - 1.08) | 0.845 |
| Robust->Pre-frail | Ever drinkers | Never drinkers | 0.97 (0.90 - 1.04) | 0.386 |
| Frail->Pre-frail | Ever drinkers | Never drinkers | 1.06 (0.95 - 1.19) | 0.287 |
| Pre-frail->Frail | Ever drinkers | Never drinkers | 0.98 (0.90 - 1.06) | 0.548 |
| Robust->Death | Ever drinkers | Never drinkers | 0.96 (0.47 - 2.00) | 0.923 |
| Pre-frail->Death | Ever drinkers | Never drinkers | 1.73 (1.03 - 2.91) | 0.038 |
| Frail->Death | Ever drinkers | Never drinkers | 0.92 (0.80 - 1.05) | 0.226 |

**Supplementary Table 4.** Effects of age × gender interaction on transition hazards in the Multi-State Markov Model (Fully adjusted for all covariates)

| **Transition** | **Covariates** | **Reference** | **HR (95% CI)** | ***p*** |
| --- | --- | --- | --- | --- |
| Robust->Pre-frail | Age × Gender Male | Age × Gender Female | 1.00 (0.99 - 1.01) | 0.929 |
| Pre-frail->Frail | Age × Gender Male | Age × Gender Female | 1.01 (1.00 - 1.01) | 0.136 |
| Pre-frail->Robust | Age × Gender Male | Age × Gender Female | 0.99 (0.98 - 1.00) | 0.002 |
| Frail->Pre-frail | Age × Gender Male | Age × Gender Female | 0.99 (0.98 - 1.00) | 0.196 |
| Robust->Death | Age × Gender Male | Age × Gender Female | 1.00 (0.93 - 1.08) | 0.975 |
| Pre-frail->Death | Age × Gender Male | Age × Gender Female | 1.03 (0.84 - 1.27) | 0.767 |
| Frail->Death | Age × Gender Male | Age × Gender Female | 0.98 (0.97 - 0.99) | 0.001 |
